# Reduced Kidney Function in Tenofovir Disoproxil Fumarate Based Regimen and Associated Factors: A Hospital Based Prospective Observational Study in Ethiopian Patients

**DOI:** 10.1155/2019/9172607

**Published:** 2019-02-03

**Authors:** Taklo Simeneh Yazie, Teferra Abula Orjino, Wondwossen Amogne Degu

**Affiliations:** ^1^Department of Biomedical Science, Samara University, Samara, Ethiopia; ^2^Department of Pharmacology and Clinical Pharmacy, Addis Ababa University, Addis Ababa, Ethiopia; ^3^Department of Internal Medicine, Addis Ababa University, Addis Ababa, Ethiopia

## Abstract

**Purpose:**

Tenofovir disoproxil fumarate (TDF), a drug broadly used in combination antiretroviral therapy, is associated with renal dysfunction but the prevalence varied from country to country and it is not known in Ethiopia. The objectives of this study were to assess the prevalence of renal dysfunction and risk factors associated with it and the mean change in estimated glomerular filtration rate in human immunodeficiency virus infected patients receiving TDF based antiretroviral regimen at Tikur Anbessa Specialized Hospital.

**Method:**

It was a hospital based prospective cohort study. The study participants were treatment naïve HIV infected patients initiating TDF containing combination antiretroviral therapy or switched to it because of adverse events. Multivariable logistic analysis was used to identify variables which have significant association.

**Result:**

A total of 63 study participants were studied, 16 (25.4%) of whom had fall in eGFR greater than 25% relative to baseline. Only age greater than 50 years, baseline CD4 count less than 200 cells/mm^3^, and baseline proteinuria were significantly associated with renal dysfunction in multivariable logistic regression. There was -8.4 ml/min/1.73m^2^ mean change in estimated glomerular filtration rate relative to baseline at six months of study.

**Conclusion:**

The renal dysfunction (defined as decline in eGFR greater than 25%) was found in a quarter of the study population. The long term impact and the clinical implication of it are not clear. Future prospective study is required with large sample size and long duration to ascertain the prevalence of decline greater than 25% in estimated glomerular filtration rate and its progression to chronic kidney disease.

## 1. Introduction

Tenofovir disoproxil fumarate (TDF) is an oral prodrug of tenofovir, a nucleotide reverse transcriptase inhibitor with activity against human immunodeficiency virus-1 and human immunodeficiency virus-2 (HIV-1 and HIV-2) [1]. It is a widely used drug in combination with other antiretroviral drugs for the treatment of HIV owing to its favorable pharmacodynamics and pharmacokinetics properties that allow once daily administration to increase adherence to lifelong treatment [2]. TDF use is generally considered safe in clinical trials [3] and a meta-analysis of 17 prospective studies (including 9 randomized controlled trials) showed that TDF based antiretroviral therapy results in a modest decline in renal function that does not restrict TDF use where regular monitoring of renal function is impractical [4]; however, there are increasing numbers of TDF induced nephrotoxicity case reports in real clinical practice [5, 6] and it has a claim to be a potential cause of both acute kidney injury (AKI) and chronic kidney disease (CKD) [7, 8]. In addition, TDF induced nephrotoxicity was reported recently in nearly 41% of participants treated with TDF based regimen for 10 years which makes its continuous use questionable [9]. The disparity of TDF safety profile between clinical trials and studies in real clinical practice may be attributed to different sociodemographic factors, presence of comorbidity, and comedications in real clinical practice [10–12]. While the exact mechanism of tenofovir nephrotoxicity remains unclear, mitochondria toxicity has been claimed as the target of tenofovir induced renal toxicity [13]. In addition, it can also indirectly damage the renal tubule possibly by activating nuclear factor kappa B protein 65 which results in inflammation induced kidney damage [14].

Several studies revealed fall greater than 25% and mean change in estimated glomerular filtration rate (eGFR) relative to baseline in TDF based antiretroviral regimen. The prevalence of fall greater than 25% in eGFR in those studies varied from country to country and it was found in the range of 6% to 40.8% [9, 15–20]. There is no study in Africa as well as in Ethiopia to show the extent of fall in eGFR greater than 25% relative to baseline. However, a prospective case cohort study in South Africa showed that among admitted patients with AKI 61% were on TDF based antiretroviral regimen [21].

Significant mean reduction in eGFR ranging from 5ml/min to 9ml/min/1.73m^2^ from baseline at 6 months of posttreatment initiation was revealed in previous studies [9, 16, 22, 23]. In contrast, studies in Africa showed a mean increase of 1.9ml/min of eGFR [24] and nonsignificant mean decline in eGFR (-0.5ml/min) [25].

Several predisposing factors were identified for TDF induced nephrotoxicity such as lower body mass index, protease inhibitor [15] low CD4 count, old age [26] and black race [27], and single nucleotide polymorphisms (SNPs) in tenofovir transporter proteins [28–30]. Highly active antiretroviral therapy adverse drug reactions in developing countries may differ from those in developed countries because of high prevalence of conditions such as malnutrition, tuberculosis, anemia, and patients presenting with advanced HIV disease [31]. Furthermore, studies indicated that black race is at increased risk of developing AKI [27, 32, 33].

Generally, the occurrence of renal dysfunction is becoming common in HIV infected patients who received TDF based antiretroviral regimen and its burden on survival and quality of life is becoming worse [34]. The condition of renal complication in HIV infected patients can be worst in low and middle income countries as the condition requires enough capital to manage renal complications. Developing countries do not have enough access to dialysis and kidney transplant procedures; this further can make the consequence of AKI worse [35].

Our national guideline put TDF based antiretroviral regimen as preferred first line for treatment of HIV in adults, adolescents, and pregnant women, and many patients are more likely to be exposed to TDF. In addition, there is no routine renal function monitoring in our clinical settings [36] and there is no local data that addresses renal dysfunction in HIV infected patients receiving TDF.

So, this study was planned to assess prevalence of renal dysfunction and risk factors associated with it and the mean change in eGFR. This study is used as a guide for early detection of renal dysfunction. It helps healthcare providers to identify HIV infected patients who are at risk. It also helps policy makers to give more attention to TDF related renal dysfunction and the necessity of future large scale study in order to know the clinical impact of TDF on renal function.

## 2. Methods

This is a prospective cohort study with 6-month duration of follow-up. Data was collected through face to face interview and by laboratory tests. Approval for the study was given by School of pharmacy and department of Internal medicine, Addis Ababa University (Ref. No. ERB/SOP/07/09/2016). Written informed consent was obtained from all study participants and for data analysis patient details were anonymized.

### 2.1. Study Participants

Participants had been recruited and enrolled prospectively before they started taking TDF based antiretroviral regimen from January 15 to March 23, 2017, Gregorian calendar. This recruitment and enrollment period was selected conveniently due to time and budget constraints. Individuals who had the following characteristics were enrolled consecutively: (1) Individuals who were voluntarily participated in the study; (2) age ≥ 18 years; (3) treatment naïve patients who were assigned to start taking TDF based antiretroviral regimen after enrollment; (4) treatment experienced patients whose antiretroviral therapy is going to be switched to TDF based antiretroviral regimen; (5) patients who had eGFR by CKD EPI equation greater than 60ml/min/1.73m^2^; (6) patients who gave consent to complete the study follow-up period. In contrast, pregnant women, inpatient individuals, and individuals who took TDF based antiretroviral regimen previously were excluded.

### 2.2. Measurement of Weight and Height

Weight and height were measured by using Seca 761 weight scales and height ruler (with meter reading) which is attached with it, respectively (made in Germany). Body mass index of participants was calculated as follows: body mass index = weight (in kg) ÷ (height (in m))^2^.

### 2.3. Measurement of Serum Creatinine and Urinalysis

After collection of urine by 30ml urine cap (HENSO Medical, Co., Ltd., China), baseline proteinuria and glycosuria were determined by comber 10 strip (HENSO Medical, Co., Ltd., China). Serum creatinine was analyzed prospectively at baseline, then after 1 month and 2 months, and at the end of 6 months by chemistry analyzer (HITACHI 902) at TASH chemistry laboratory.

The selection of each study visit (1, 2, and 6 months) for the determination of eGFR was based on previous studies [15, 16, 18].

### 2.4. eGFR Measurements

Renal dysfunction was defined as more than 25% decline in eGFR relative to baseline after commencement of TDF based antiretroviral regimen [17]. Guideline and study recommend the use of CKD EPI equation to calculate eGFR in HIV infected patients against other equations [37, 38]. Serum creatinine values were used to calculate eGFR in the following equations.

- For female with serum creatinine ≤ 0.7mg/dl: GFR = 166 × (Scr/0.7)^−0.329^ × (0.993)^Age^; female with serum creatinine > 0.7mg/dl: GFR = 166 × (Scr/0.7)^−1.209^ × (0.993)^Age^
- For male with serum creatinine ≤ 0.9mg/dl: GFR = 163 × (Scr/0.9)^−0.411^ × (0.993)^Age^; female with serum creatinine > 0.9mg/dl: GFR = 163× (Scr/0.9)^−1.209^ × (0.993)^Age^ [39].
- Here, age is in year and serum creatinine is in mg/dl.

### 2.5. Data Analysis

Mean (±standard deviation (SD)), median (interquartile range (IQR)), frequencies, and percent (%) were used to describe patients' characteristics. The prevalence of decline in eGFR greater than 25% relative to baseline was calculated by dividing a number of patients with decline in eGFR greater than 25% by total number of patients and multiplying by 100. A repeated measures (within-subjects) analysis of variance (ANOVA) was used to compare means of eGFR obtained at different study visits and post hoc tests were performed by paired* t*-test using Bonferroni correction to pinpoint specific mean that significantly differed from other means. Univariate logistic regression was used to determine the factors associated with renal dysfunction. Clinically significant factors in the literature were entered in multivariable logistic regression without restriction by p < 0.2. Other independent variables that presented with P < 0.20 were considered in a multivariable logistic regression model. Adjusted odds ratio (AOR) and its 95% CI were estimated. A p value <0.05 was considered statistically significant. All statistical analyses were performed using version 20 of SPSS program.

## 3. Result

### 3.1. Sociodemographic Characteristics of Study Participants

A total of 66 HIV infected patients with 61 treatment naïve patients to antiretroviral therapy and 5 treatment experienced patients whose antiretroviral therapy is going to be switched to TDF based antiretroviral therapy were enrolled in the study. Three treatment naïve participants were lost to follow-up without having serum creatinine values after baseline visits and nonadherence was reported as the reason for their loss of follow-up. The age of lost participants was around the median age of study participants. Among lost participants, 2 had CD4 counts lower than the median CD4 count of the study participants and 1 had CD4 count higher than the median CD4 count of participants. A total of 63 participants were included in the final analysis. The mean (± SD) age was 39.7 (±10); 43 (68.3%) of study participants were female. The mean (±SD) body mass index was 22.6 (±4.5) kg/m^2^ and other sociodemographic characteristics are shown in Table 1.

### 3.2. Clinical Characteristics of Study Participants

Among participants, 5 (7.9%) had prior exposure to zidovudine based antiretroviral regimen. Majority of patients (56, 88.9%) were taking TDF + lamivudine + efavirenz whereas the remaining patients were taking TDF + lamivudine+ ritonavir boosted atazanavir regimen. Median (IQR) CD4 count was 241 (106-457); 42 (66.7%) of patients were found in World Health Organization (WHO) clinical stage I. The median (IQR) of duration of HIV infection since diagnosis was 3 months (0 – 60); 20 (31.8%) of study participants were with comorbidity of either hypertension (6.4%), type 2 diabetes mellitus (3.2%), cancer (12.7%), tuberculosis (12.7%), or kidney stone (3.2%). Opportunistic infections other than tuberculosis were found in 8 (12.7%) of participants.

Among study participants, 8 (12.7%) were taking isoniazid preventive therapy. In addition, angiotensin converting enzyme inhibitor (enalapril) + hydrochlorothiazide and metformin + glibenclamide were taken by 4 (6.3%) and 2 (3.2%) of study participants, respectively, and other clinical characteristics are shown in Table 2.

### 3.3. Renal Dysfunction among Study Participants

Among study participants fall in eGFR greater than 25% was found in 16 (25.4%) of study participants during the entire study period and the majority of these case occurred in the first month of study follow-up period. Out of 16 (25.4%) of study participants who were diagnosed with renal dysfunction, 7 (11.1%) and 9 (14.3%) were male and female, respectively. Renal dysfunction occurred in 15 (23.8%) of participants who were taking TDF + lamivudine + efavirenz and in 1 (6.2%) of participants who were taking TDF + lamivudine + ritonavir boosted atazanavir regimen. In the study period, 11 (93.3%) of the cases occurred in patients who had baseline eGFR equal to or greater than 90ml/min/1.73m^2^ (Table 3).

In the current study, chronic kidney disease (CKD) was confirmed by 2 consecutive measurements of eGFR < 60ml/min/1.73m^2^ at 4-month interval and CKD was detected in 2 (3.2%) of study participants (Table 4).

At the end of 6 months, the prevalence of proteinuria was higher than the prevalence of baseline proteinuria (27% and 20.6%, respectively). Among 27% of proteinuria, 14.3% of proteinuria was found in patients with renal dysfunction. However, the prevalence of glycosuria at the end of 6 months was the same as the prevalence of baseline glycosuria (4.8%).

### 3.4. Factors Associated with Renal Dysfunction

Hypertension, type 2 diabetes mellitus, tuberculosis, kidney stone, and prior exposure to antiretroviral drugs were not included in univariate logistic analysis because participants with these factors did not experience renal dysfunction. Clinically significant factors (body mass index, chemotherapy, age, and protease inhibitor) were included in multivariable logistic regression without restriction by p < 0.2. Independent variables which were entered in univariate logistic regression were sex, ethnicity, age, body mass index, WHO clinical stage, baseline CD4 count, duration of HIV infection since diagnosis, current ART, cotrimoxazole prophylaxis therapy, isoniazid preventive therapy, history of cancer, presence of opportunistic infections, presence of chemotherapy, proton pump inhibitors, baseline proteinuria, baseline glycosuria, baseline serum creatinine, and baseline eGFR. Among them, baseline CD4 count, baseline eGFR, baseline proteinuria, history of cancer, and cotrimoxazole prophylaxis therapy were significantly associated with renal dysfunction.

In multivariable logistic regression, age greater than 50 years, baseline proteinuria, and baseline CD4 count less than 200 cells/mm^3^ were significantly associated with renal dysfunction (AOR = 64.8, 95% CI 1.60-2707.70, and P = 0.029; AOR = 51.3, 95% CI 1.80-1448.70, and P = 0.021; AOR = 63.2, 95% CI 2.02-1979.66, and p = 0.018), respectively. However, other variables did not maintain their statistical significance in multivariable logistic regression (Table 5).

### 3.5. Mean Change in Estimated Glomerular Filtration Rate

The mean (± SD) baseline eGFR of study participants was 90.8 (± 16.8)ml/min/1.73m^2^ and 55.6% of them had baseline eGFR of less than 90ml/min/1.73m^2^. A repeated measures one-way ANOVA determined that means differed significantly between time points (F (2.63, 163.32) = 8.80, P < 0.005). Post hoc tests using Bonferroni correction revealed nonsignificant mean increase of eGFR from post-1-month to post-2-month TDF based regimen initiation (p = 1) whereas there was nonsignificant mean reduction of eGFR from post-1-month to post-6-month (P = 1) and from post-2-month to post-6-month TDF based regimen initiation (P = 1). Post hoc tests using Bonferroni correction showed significant mean reduction of eGFR from baseline to the case after 1, 2, and 6 months of TDF based regimen initiation.

A repeated measures one-way ANOVA confirmed that means of SCr significantly differed between time points (F (2.75, 170.33) = 8.58, P < 0.0005). Post hoc tests using Bonferroni correction revealed that there was no significant difference between mean of SCr after 1 month and 2 months, after 1 month and 6 months, and after 2 months and 6 months of TDF based regimen initiation. Post hoc tests using Bonferroni correction showed significant mean increase of SCr from baseline to the case after 1, 2, and 6 months of TDF based regimen initiation (shown in Table 6).

## 4. Discussion

In the present study, renal dysfunction was detected in 16 (25.4%) of study participants and factors associated with renal dysfunction were age greater than 50 years, baseline CD4 count less than 200 cells/mm^3^, and baseline proteinuria. In this study, there was significant mean reduction of eGFR at 1, 2, and 6 months of post-TDF based regimen initiation compared to mean baseline eGFR (-8.35; P = 0.001, -7.89; P = 0.001 and -8.44; P = 0.002, respectively).

Among participants who were diagnosed with renal dysfunction at end of first-month visit, 9.5% of them continued with renal dysfunction at 2- and 6-month study visits. However, none of them developed CKD. The prevalence of renal dysfunction in the current study was higher than from studies conducted in Thailand (19.3%), Malaysia (15.2%), Japan (19.6%, 22.1%), Spain (10%), Vietnam (12.4%), and Korea [15–20, 26]. This discrepancy may be attributed to difference in genetic factors as some patients experience renal adverse effects of tenofovir more frequently than others. This issue may be related to genetic polymorphism in drug transporter proteins of the renal tubule and consequently accumulation of TDF in proximal tubular cells may lead to reduction in glomerular filtration rate [40]. Participants in Thailand, Spain, Vietnam, and Korea had higher median baseline CD4 count (more than 320 cells/mm^3^) than the median CD4 count (241 cells/mm^3^) of the participants of the present study. Participants in Japan and Vietnam had younger median age (36 years) than participants (40 years) of the present study. Therefore, these differences in median CD4 count and age may partially explain the discrepancy between the results of the current study and the previous studies.

The finding of the present study was lower than the finding of another study done in Japan (40.8%) [9]. This discrepancy may be attributed to difference in sociodemographic factors and number of study participants. A study done in Japan was a 10-year follow-up study and most of patients (89%) compared to this study (11.1%) were taking protease inhibitor based antiretroviral regimen which is known to decrease eGFR greater than TDF regimen with nonnucleoside reverse transcriptase inhibitors [15]. Therefore, these differences in duration of follow-up and proportion of participants taking protease inhibitor based antiretroviral regimen may be another possible reason for the variation of the findings.

Even if assessment of CKD was not the objective of the present study, CKD was diagnosed in 2 (3.2%) of participants which was similar to another study [41]. The result of this study was lower than the result of the study done in Japan. The difference might be due to difference in duration of study follow-up [42].

The result of this study was lower than the results of the studies done in Africa. However, studies in Africa were cross-sectional which diagnosed CKD at a point of time which might overestimate CKD [38, 43]. In addition, the prevalence of APOL1 risk variants for renal disease was found to be low among Ethiopians compared to other Africans. Therefore, this can be considered as additional explanation for the disagreement of the findings [44]. The finding of this study was higher than the study finding in Italy. Participants in Italy did not receive protease inhibitors and had higher median baseline CD4 count, so these differences might be the reason for the discrepancy of the findings [22]. The difference also might be attributed to sociodemographic factors because black race is more risky for developing CKD [27].

In the present study, age greater than 50 years was associated with renal dysfunction (AOR = 64.8, 95% CI 1.60-2707.70, and p = 0.029). This finding was in line with another finding that was done in Malaysia [26] and it is known that age greater than 50 years is an established risk factor for tenofovir induced nephrotoxicity. This can be explained by age related structural and physiological deterioration of the kidney [8].

There was a significant association between baseline CD4 count less than 200 cells/mm^3^ and eGFR fall greater than 25% as compared to study participants who had baseline CD4 count equal to or greater than 200 cells/mm^3^ in the present study (AOR = 63.2, 95% CI 2.02-1979.66, and P = 0.018). This result was similar to the study in Maryland and United States of America [3, 11]. Study done in Spain also revealed that patients who had lower baseline CD4 count were associated with AKI [18]. This might be related to the fact that patients with advanced HIV infection are more risky to renal dysfunction owing to direct injury of HIV on renal cells [10]. However, the occurrence of HIV associated nephropathy is less likely in the present study because Ethiopians have low prevalence of APOL1 risk variants [44].

The present study had also shown that there was association between baseline proteinuria and renal dysfunction (AOR = 51.3, 95% CI 1.80-1448.70, and P = 0.021) and this result was similar to study conducted in Canada [45]. The study done in Japan also found that participants with proteinuria had significantly low eGFR compared to participants without proteinuria [46]. In the current study body mass index was not associated with renal dysfunction in contrast to the finding of the study in Thailand (AOR = 2.26) [15]. The discrepancy might be attributed to difference in number of study participants.

The present study by post hoc tests using Bonferroni correction showed significant mean reduction of eGFR from baseline to the case after 1, 2, and 6 months of TDF based regimen initiation. The mean decline in eGFR of -8.4ml/min/1.73m^2^ at the end of the study relative to baseline was similar to other studies [9, 16, 23]. This result was higher than the finding of the study done in Italy. The participants of the study in Italy had higher median baseline CD4 count than participants of this study and no participants received protease inhibitors. So, these differences might be the reason for the disagreement of the findings [22].

In addition, the finding of the current study was higher than results from study done in Africa (participants recruited from Senegal and Cameroon). Participants of the study in Africa did not take protease inhibitors and had no comorbidities in contrast to our study. These differences might partially explain the discrepancy of the findings [24]. The result of the study in Africa showed a mean increase of 1.9ml/min in eGFR, which is in contrast to findings from several studies [9, 16, 23]. How tenofovir increases glomerular filtration rate in African HIV infected patients is not yet clear.

The result of the current study was also higher than the result of the study from South Africa. The discrepancy of the findings might be partially due to difference in inclusion criteria. The median age of South African participants was 35.4 years whereas median age of the participants of this study was 40 years [25]. The mechanism by which tenofovir reduces glomerular filtration rate is not well understood. In fact, tenofovir is known to cause renal tubular dysfunction [29], which might subsequently result in significant reduction in glomerular filtration rate [30, 46].

The present study has strong side of being the first, prospective cohort study in Ethiopia. In this study a quarter of participants were diagnosed with renal dysfunction, which makes the long term use of TDF questionable. However, this study has limitations of small sample size and relatively short duration of follow-up. In addition, renal tubular dysfunction was not assessed in this study, so tenofovir associated renal dysfunction may be underestimated.

In conclusion, the current study demonstrated that fall in eGFR greater than 25% occurred in a quarter of participants. Age greater than 50 years, CD4 count less than 200 cells/mm3, and baseline proteinuria were risk factors for the occurrence of renal dysfunction.

## Figures and Tables

**Table 1:** Baseline sociodemographic characteristics of study participants in Tikur Anbessa Specialized Hospital (TASH), October 2017 [n = 63].

Characteristics		Frequency (%)
*Sex*	Male	20 (31.7)
	Female	43 (68.3)
*Marital status*	Single	8 (12.7)
	Married	40 (63.5)
	Divorced	11 (17.5)
	Widowed	2 (3.2)
	Missing	2 (3.2)
*Educational status*	Illiterate	5 (7.9)
	Primary	18 (28.6)
	Secondary	28 (44.4)
	Diploma	5 (7.9)
	Degree	7 (11.1)
*Ethnicity*	Oromo	20 (31.7)
	Amhara	22 (34.9)
	Tigre	6 (9.5)
	Others	15 (23.8)
*Occupation*	governmental employee	12 (19)
	private	34 (54)
	not employed	17 (27)
*Age*	≤50 years old	56 (88.9)
	>50 years old	7 (11.1)
*Body mass index (in kg/m* ^*2*^)	<18.5	12 (19)
	≥18.5	51 (81)

Note: other ethnic groups include mixed ethnicity, Gurage, Silte, Hadiya, Welayta, and Harari.

**Table 2:** Baseline clinical characteristics of study participants in TASH [n = 63].

Characteristics		Frequency (%)
*WHO stage*	Stage 1	42 (66.7)
	Stage 2	3 (4.8)
	Stage 3	6 (9.5)
	Stage 4	12 (19)
*Cotrimoxazole prophylaxis*	Yes	42 (66.7)
	No	21 (33.3)
*Duration of HIV infection since DX*	Median (IQR) 3 (0-60) months	
	< 3 months	37 (58.7)
	≥3 months	26 (41.3)
*Baseline CD4 count (cells/mm* ^*3*^)	Median (IQR) 241 (106-457)	
	< 200 CD4 count	29 (46)
	≥ 200 CD4 count	34 (54)
*Baseline serum creatinine (mg/dl)*	Mean (±SD) 1.01 (±0.16)	
	< 1	18 (28.6)
	≥1	45 (71.4)
*Baseline eGFR (ml/min/1.73m* ^*2*^)	Mean (±SD) 90.8 (±16.8)	
	< 90	35(55.6)
	≥90	28 (44.4)
*History of cancer*	Yes	8 (12.7)
	No	55 (87.3)
*Antituberculosis drugs*	Yes	4 (6.3)
	No	59 (93.7)
*NSAIDs*	Yes	6 (9.5)
	No	57 (90.5)
*Proton pump inhibitor*	Yes	9 (14.3)
	No	54 (85.7)
*Chemotherapy*	Yes	4 (6.3)
	No	59 (93.7)
*Baseline proteinuria*	+1	9 (14.3)
	+2	4 (6.3)
*Baseline glycosuria*	+1	2 (3.2)
	+3	1 (1.6)

WHO, World Health Organization; DX, diagnosis; IQR, interquartile range; SD, standard deviation; NSAIDs, nonsteroidal anti-inflammatory drugs; eGFR, estimated glomerular filtration rate.

**Table 3:** Greater than 25% fall in estimated glomerular filtration rate of study participants in TASH, October 2017 [n = 63].

		Greater than 25% decline in eGFR	
		New cases	Total cases
*Months*	0	-	-
	1	10 (15.9)	10 (15.9)
	2	2 (3.2)	9 (14.3)
	6	4 (6.3)	13 (20.6)

Note: glomerular filtration rate was estimated by CKD EPI equation and events expressed in number (percent); eGFR, estimated glomerular filtration rate in ml/min/1.73m^2^. New cases mean cases that occurred newly at each visit. Total cases mean sum of cases that occurred in previous study visit (that persisted) and new cases that occurred at a given visit.

**Table 4:** Chronic kidney disease among study participants in TASH, October 2017 [n = 63].

		eGFR < 60ml/min/1.73m^2^ measured at 1 point of time	eGFR < 60ml/min/1.73m^2^ measured at 4-month interval
		Number (%)	Number (%)
*Months*	0	0 (0)	-
	1	7 (11.1)	-
	2	4 (6.3)	-
	6	3 (4.8)	2 (3.2)

**Table 5:** Factors associated with greater than 25% fall in eGFR during the study period by univariate and multivariable logistic regression in TASH, October 2017 [n = 63].

Variable	Category	Renal dysfunction		Univariate COR (95% CI)	Multivariable AOR (95% CI)
		Yes	No		
*Sex*	Male	7	13	1.0	1.0
	Female	9	34	0.5 (0.20, 1.64)*∗*	19.0 (0.30, 1338.12)
*Age*	≤ 50 years	13	43	1.0	1.0
	> 50 years	3	4	2.5 (0.49, 12.54)*∗*	64.8(1.60, 2707.70)*∗∗*
*Body mass index (kg/m* ^*2*^)	< 18.5	3	9	1.0 (0.23, 4.16)	0.08 (0.01, 3.40)
	≥18.5	13	38	1.0	1.0
*Baseline CD4 count*	< 200 CD4 count	13	16	8.4 (2.10, 33.80)*∗*	63.2(2.02, 1979.66)*∗∗*
	≥200 CD4 count	3	31	1.0	1.0
*Current ART*	TDF+3TC+EFV	15	41	1.0	1.0
	TDF+3TC+ATV/r	1	6	0.5 (0.50, 4.10)	0.1 (0.01, 32.90)
*Cotrimoxazole prophylaxis*	Yes	15	27	11.1 (1.40, 92.10)*∗*	0.91 (0.04, 22.43)
	No	1	20	1.0	1.0
*History of cancer*	Yes	5	3	6.7 (1.40, 32.30)*∗*	36.6 (0.50, 2699.88)
	No	11	44	1.0	1.0
*Chemotherapy*	Yes	2	2	3.2 (0.40, 25.00)	3.74 (0.02, 938.60)
	No	14	45	1.0	
*Baseline serum creatinine*	< 1mg/dl	7	11	2.6 (0.80, 8.40)*∗*	0.35(0.01, 9.90)
	≥ 1mg/dl	9	36	1.0	1.0
*Baseline eGFR (ml/min/1.73m* ^*2*^)	< 90	5	30	1.0	1.0
	≥ 90	11	17	3.9 (1.15, 13.06)*∗*	11.6(0.23, 587.30)
*Proteinuria*	Yes	8	5	8.4 (2.20, 32.30)*∗*	51.33(1.80, 1448.70)*∗∗*
	No	8	42	1.0	1.0
*Presence of glycosuria*	Yes	2	1	6.6(0.60, 78.00)*∗*	53.0 (0.49, 5774.33)
	No	14	46	1.0	1.0

COR, crude odds ratio; AOR, adjusted odds ratio; CI, confidence interval; ART, antiretroviral therapy; 3TC, lamivudine; EFV, efavirenz; ATV/r, ritonavir boosted atazanavir. *∗* indicates variables that were entered in univariate logistic regression; *∗∗* indicates variables which were significant in multivariate logistic regression (P < 0.05).

**Table 6:** Changing patterns of estimated glomerular filtration rate and serum creatinine over 6-month follow-up of study participants in TASH, October 2017 [n = 63].

	Months			
	0	1	2	6
*Mean eGFR ± SD*	90.81 ± 16.8	82.46 ± 17	82.93 ± 15.3	82.44 ± 15
*Mean change in eGFR ± SD* *95*%* CI of mean change in eGFR*	-	-8.35 ± 17.5*∗*^+^	-7.89 ± 15.10*∗*^+^	-8.37 ± 18.4*∗*^+^
	-	-12.81, -3.8	-11.70, -4.10	-13.00, -3.70
*Mean SCr ± SD*	1.01 ± 0.16	1.10 ± 0.19	1.09 ± 0.17	1.10 ± 0.17
*Mean change in SCr ± SD* *95*%* CI of mean change in SCr*	-	0.09 ± 0.18*∗*^+^	0.08 ± 0.17*∗*^+^	-0.08 ± 0.19*∗*^+^
	-	0.05, 0.14	0.04, 0.12	0.04, 0.13

Note: eGFR, estimated glomerular filtration rate; SEM, standard deviation; CI, confidence interval; SCr, serum creatinine. P values by repeated measures one-way ANOVA (*∗* stands for P < 0.05) and post hoc tests using paired *t*-test with Bonferroni correction (^+^ stands for P < 0.05).

## Data Availability

All data used for the current study are available from the corresponding author on justifiable request.
